# Excision of the Superior Maxilla, with Simultaneous Ligation of the External Carotid

**Published:** 1890-08

**Authors:** Wm. Perrin Nicolson

**Affiliations:** Atlanta, Ga.


### EXCISION OF THE SUPERIOR MAXILLA, WITH SIMULTANEOUS LIGATION OF THE EXTERNAL CAROTID.

---

BY WM. PERRIN NICOLSON, M. D., ATLANTA, GA.

---

On May 23rd, 1890, I saw at the request of Dr. Stewart, of Conyers, Ga., Mrs. A., with a history of a tumor of the upper jaw of about one year's duration. It was described as beginning as a "gum-boil," and increasing slowly, until pregnancy supervened, and during the last months the increase had been very rapid. At my visit I learned that her confinement had taken place just a week before, and on account of the increasing size of the tumor she and her physician were anxious for an immediate operation. She was unable, and had for sometime been, to take any solid food, as the encroachment of the tumor on the roof of the mouth rendered her unable to chew. Recognizing the growth as malignant, and almost certain to speedily return, I concluded to make a ligation of the external carotid artery, in the hope that the cutting off of the nutrition from its area of distribution might in a measure retard its re-appearance. The vessel was reached and tied at a point half an inch above the bifurcation, and the wound closed without drainage, catgut being used both for the ligature and sutures. The effect of the closure of the vessel was interesting. The face being flushed from the anæsthetic, as a first effect one half was blanched, but by the time the wound in the neck was closed, the blood from the other side had crept over, and there was no distinguishable difference in the two sides. The face wound oozed profusely at first, but the bone was extirpated without the ligation of a single vessel, and after closing it the cavity was stuffed with iodoform gauze.

Recovery was uninterrupted, and on the eighth day the patient was out of the house. Both wounds healed by primary union, and the cutting off of the blood supply seemed to have no effect on the healing process.

Professor Bryant, in a paper read before the New York State Medical Association, and published in the Annals of Surgery, calls attention to the fact that the ligation of the external carotid artery in such cases is justifiable, both as a step looking to the prevention of hemorrhage and lessening the danger of a return of the growth.

Dr. Wyeth has done much to prove that the ligation of the vessel is comparatively free from danger, certainly as compared with the statistics of ligation of the common carotid.

Subsequent examination of the specimen shows it to be a small, round-celled sarcoma.

